# Friendship and Loneliness Among People Living With Dementia: Toward Community and Shared Humanity

**DOI:** 10.1093/geroni/igab046.1533

**Published:** 2021-12-17

**Authors:** Daniel R Y Gan, Habib Chaudhury, Jim Mann

## Abstract

An increasing number of people living with dementia (PLWD) age in community. In North America, this number ranges from 61-81% of the total number of PLWD. As many as one in three PLWD do not live with a care partner. Since most PLWD do not drive, many may spend a significant proportion of time within half a mile of their homes. Yet, the neighbourhood may or may not provide “ways of being in the world that are more accepting and embracing of the kinds of disruptions that dementia can produce” (Hillman & Latimer, 2017). To support continued social participation, meaningful everyday networks are required. PLWD and care partners may identify more or less strongly with a community depending on their position in the network, its spaces, and activities. According to Nancy (1991), “community” has been conjured as an antidote to the loneliness of the human condition, which explains its allure. In response, Costello (2014) argued that “community” requires one to constantly try and “fall short” in caring for another’s changing experiences. The value of a community thus depends on the quality of its friendships – the ability of otherwise lonely individuals to empathize – which may be threatened by challenges to PLWD’s personhood. This symposium brings together expertise in community gerontology, philosophy, and neuropsychology to advance current conceptualizations of personhood in community amid cognitive decline. These will be discussed in relation to lived experiences, with the aim to inform future research and practice of dementia care and prevention in community.

